# Influence of gender and age on the survival of patients with nasopharyngeal carcinoma

**DOI:** 10.1186/1471-2407-13-226

**Published:** 2013-05-04

**Authors:** Guangli Xiao, Yabing Cao, Xibin Qiu, Weihua Wang, Yufeng Wang

**Affiliations:** 1 Radiation Therapy Center, Kiang Wu Hospital, Macao, China

**Keywords:** NPC, Gender, Age and survival

## Abstract

**Background:**

The prognostic value of gender and age in the survival of nasopharyngeal carcinoma (NPC) patients treated with intensity-modulated radiotherapy (IMRT) is unclear. Several studies have suggested a female advantage in the prognosis of solid tumors. We investigated the relationship between gender differences and disease outcome in NPC patients treated with IMRT in South China.

**Methods:**

A total of 299 patients with non-disseminated NPC were analyzed retrospectively. IMRT was delivered with a simultaneous modulated, accelerated radiotherapy boost technique at prescribed doses of 70 Gy/30 fractions/6 weeks to the primary tumor (GTVp) and positive neck nodes (GTVn), 60Gy (2.0 Gy/day) to the clinical target volume (CTV) and upper neck region and 54 Gy (1.8 Gy/day) to the clinically negative low neck. A median boost dose of 9.2 Gy (4–20 Gy) was administered to patients with persistent disease at the primary site.

**Results:**

With a median follow-up of 52 months, the male patients had a significantly unfavorable 5-year OS (70.7% compared to 94.1%, P < 0.001), DPFS (71.5% compared to 87.3%, P = 0.029) and DMFS (77.2% compared to 89.7%, P = 0.036) than the female patients. In patients younger than 45, the male patients had a poorer 5-year OS (66.8% compared to 91.2%, P = 0.008), DPFS (59.9% compared to 91.2%, P = 0.005) and DMFS (66.4% compared to 94.0%, P = 0.004) than the female patients. For patients older than 45, only the 5-year OS (72.2% compared to 96.0%, P = 0.001) was significantly different.

**Conclusions:**

Gender and age are strong independent prognostic factors for NPC in this study. We are the first to report that younger male patients were more likely to have distant metastases and exhibited inferior overall survival and disease progression-free survival rates compared to other patients.

## Background

The epidemiology of nasopharyngeal carcinoma (NPC) shows a uniquely skewed geographic distribution. High incidence rates are observed in the southern part of China. According to the registry data from Macao in 2009, NPC was the fifth most common cancer in men and ninth most common cancer in women. The incidence and mortality rates of NPC were 15.5 and 11.3, respectively, for men per 100,000 people and 5.4 and 0.4, respectively, for women per 100,000 people. NPC was also the most common cancer in young male adults, accounting for 23% of all cancers in that age group [1]. It is unclear why male patients displayed higher morbidity and mortality. Many studies on the prognostic factors for NPC have been published. A major limitation of the previous studies examining the effect of gender and age is the lack of adequate adjustment for other relevant clinical prognostic factors. IMRT has been used in our center since 2003, and we have treated more than 400 patients with NPC using IMRT. In this article, we explore the prognostic factors associated with these patients.

## Methods

### Patient characteristics

Between December 2003 and November 2010, there were 314 consecutive, newly diagnosed NPC patients in our Hospital. Of these patients, 13 patients with distant metastases before treatment or within 3 months after the completion of radiation therapy were excluded, 1 patient was excluded for emotional reasons, and 1 patient who died from another disease during treatment was excluded. The remaining 299 patients were included in the present study.

There were 213 (71.2%) male and 86 (28.8%) female patients. The median age was 49 (range 18 to 83). According to the AJCC/UICC (7th edition) staging criteria, there were 8 patients (2.7%) with stage I disease, 29 patients (9.7%) with stage II disease, 190 patients (63.5%) with stage III disease and 72 patients (24.1%) with stage IV disease. Thus, a total of 262 (87.6%) patients presented with stage III-IV cancer. All patients were histologically confirmed cases: there were 266 (89.0%) undifferentiated carcinomas (Grade 4), 28 (9.4%) poorly differentiated carcinomas (Grade 3) and 5 (1.7%) moderately differentiated carcinomas (Grade 2).

### Radiotherapy

All patients received an entire course using the intensity modulated radiotherapy technique (IMRT). The primary nasopharynx and the upper neck were treated with IMRT using coplanar beams. The lower neck and the supraclavicular fossae were irradiated with a single anterior field using conventional radiotherapy. The gross tumor volume (GTV) was defined as all gross disease detected by the imaging studies and physical examination and included the primary tumor (GTVp) and all enlarged neck nodes (GTVn). We designed two planning target volumes (PTV1 and PTV2). Planning target volume 1 (PTV1) encompassed the GTVp with a 5–10 mm margin and GTVn with a 2–3 mm margin of adjacent tissue, ensuring that the high-dose zone would irradiate the tumor. Planning target volume 2 (PTV2) consisted of the clinic target volume (CTV) and included the entire nasopharynx, retropharyngeal lymph node regions, parapharyngeal space, posterior nasal cavity, skull base, clivus, inferior sphenoid sinus and bilateral upper deep jugular nodes, with a margin to account for patient motion and setup error. If the level I area lymph node was involved or the bulky IIa lymph node was at presentation, the field was required to cover the level I area. The surrounding critical normal structures such as the brainstem, spinal cord, optic nerves, chiasm, lens, parotid glands, temporomandibular joints, temporal lobe, and inner ears were also outlined.

The prescribed dose for the GTV, PTV1 and PTV2 were 70 Gy, 66 Gy and 60 Gy, respectively, to at least 95% volume of the targets in 30 fractions over 6 weeks. The anterior cervical photon field delivered 66 Gy to the visible neck nodes plus a 2 mm margin, as well as 54 Gy to the clinically negative nodal regions. Persistent disease in the primary site was given a median boost dose of 9.2 Gy (4–20 Gy) with IMRT or X-knife.

### Chemotherapy

Concurrent chemoradiation was prescribed for 178 (59.5%) patients with a heavy tumor burden, who were treated with weekly cisplatin (40 mg/m^2^) during the course of RT. There were 21 patients who received neoadjuvant chemotherapy, such as cisplatin or carboplatin plus docetaxel, whereas 10 patients were given adjuvant chemotherapy because of a large tumor burden or a poor response to RT. The decision to treat with chemotherapy was made by each patient’s physician.

### Follow-up and statistical analysis

The follow-up duration was calculated from the first date of treatment; the cut-off date was the end of March 2012 or the time of death. Among living patients, all had a follow-up time of at least 22 months.

The data were analyzed using the SPSS 17.0 statistical software package. Survival rates were estimated with the Kaplan-Meier method. Differences in survival curves between the subgroups were analyzed using the log-rank test. Multivariate analysis was performed using the Cox regression model. Correlation analysis was conducted using bivariate correlation.

## Results

With a median follow-up of 52 months (range 5 to 99 months), there were 18 (6.0%) patients who developed local failure, including 1 patient (3%) with T1 stage, 3 patients (4.5%) with T2 stage, 8 patients (5.5%) with T3 stage and 6 patients (11.3%) with T4 stage. The median time of local failure was 30 (9–96) months. There were 12 (4.0%) patients who developed regional nodal failure. The median time of regional failure was 28 (6–63) months. Fifty-five (18.4%) patients developed distant metastases. The median time of distant failure was 21 (5–78) months.

The gender ratio was balanced for age, disease staging, lymph node metastasis characteristics (tumor size, necrosis and extracapsular extension), treatment methods and radiation reaction. Although it seems that the proportion of advanced cases is higher in male than female patients, the difference is not statistically significant. The median time of therapeutic failure was significantly different between male and female patients (Table 1).

**Table 1:** The characteristics and prognosis for male and female patients

**Variable**	**Men**	**Women**	**P value**
Patient (%)	213 (71.2)	86 (28.8)	
Age (year)	49 (18–83)	47 (22–75)	0.515
Chemotherapy	123 (57.7)	52 (60.5)	0.835
Histologic type			
undifferentiated carcinoma	185 (86.9)	81 (94.2)	0.680
poorly differentiated carcinoma	25 (11.7)	3 (3.5)	
moderately differentiated carcinoma	3 (1.4)	2 (2.3)	
nasopharynx tumor volume (cm^2^) (median)	36.5 (1.2-279.3)	27.2 (1.5-222.9)	0.055
Lymph node max diameter (cm) (median)	2.5 (0.5-9.0)	2 (0.4-13.0)	0.253
T stage			
T1	22 (10.3)	11 (12.8)	0.563
T2	47 (22.1)	20 (23.3)	
T3	106 (49.8)	40 (46.5)	
T4	38 (17.8)	15 (17.4)	
N stage			
N0	11 (5.2)	5 (5.8)	0.166
N1	37 (17.4)	22 (25.6)	
N2	145 (68.1)	53 (61.6)	
N3	20 (9.4)	6 (7.0)	
TNM stage			
stage	4 (1.9)	4 (4.7)	0.192
stage	18 (8.5)	11 (12.8)	
stage	139 (65.3)	51 (59.3)	
stage	52 (24.4)	20 (23.3)	
Local failure (%)	13 (6.1)	5 (5.8)	0.924
Regional failure (%)	11 (5.2)	1 (1.2)	0.111
Distant metastasis (%)	45 (21.1)	10 (11.6)	0.055
Tumor progression (%)	57 (26.8)	14 (16.3)	0.054
Median time to local failure (m)	22 (9–93)	49 (10–96)	0.017
Median time to regional failure (m)	26 (6–63)	41	0.015
Median time to distant metastasis (m)	19 (5–78)	23 (7–73)	0.020
Median time to disease progression (m)	18 (5–93)	27.5 (7–96)	0.010

**Figure 1:**
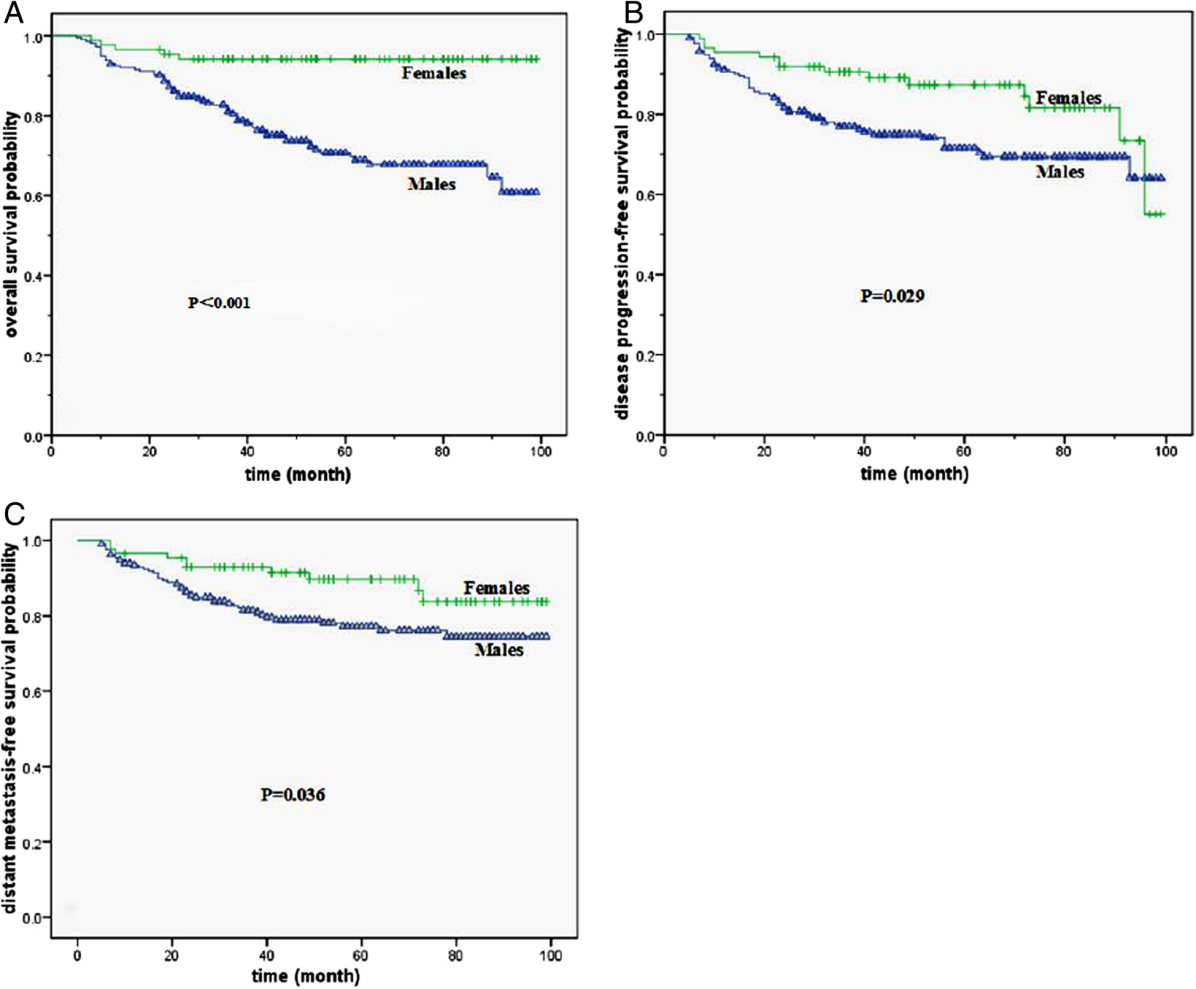
**Survival curves for male and female patients. A.** The overall survival curves of male and female. **B.** The disease progression-free survival curves of male and female. **C.** The distant metastasis-free survival curves of male and female.

Of all the patients, the male patients had a significantly unfavorable 5-year overall survival (OS) (70.7% compared to 94.1%, P < 0.001, *X*^2^ = 16.816), disease progression-free survival (DPFS) (71.5% compared to 87.3%, P = 0.029, *X*^2^ = 4.743) and distant metastasis-free survival (DMFS) (77.2% compared to 89.7%, P = 0.036, *X*^2^ = 4.383) compared to female patients (Figure 1). The number of local or regional failures was too small to allow meaningful analysis, and the 5-year local progression-free survival (LPFS) (92.9% compared to 95.6%, P = 0.684, *X*^2^ = 0.165) and regional progression-free survival (RPFS) (93.6% compared to 98.4%, P = 0.084, *X*^2^ = 2.964) were not different between genders.

For patients with stage I-II, stage III and stage IV cancers, the 5-year OS between male and female patients was 94.7% compared to 100% (P = 0.182, *X*^2^ = 1.784), 77.8% compared to 96.1% (P = 0.009, *X*^2^ = 6.916) and 41.6% compared to 84.4% (P = 0.006, *X*^2^ = 7.442), respectively. The differences in the 5-year DPFS and DMFS between male and female with stage I-II, stage III and stage IV were not significantly different: for DPFS the values were 100% compared to 100%, 73.6% compared to 89.1% (P = 0.170,*X*^2^ = 1.885) and 51.7% compared to 72.2% (P = 0.153, *X*^2^ = 2.046), respectively for DMFS the values were 100% compared to 100%, 79.1% compared to 91.2% (P = 0.132, *X*^2^ = 2.269) and 58.5% compared to 76.2% (P = 0.232, *X*^2^ = 1.430), respectively.

**Figure 2:**
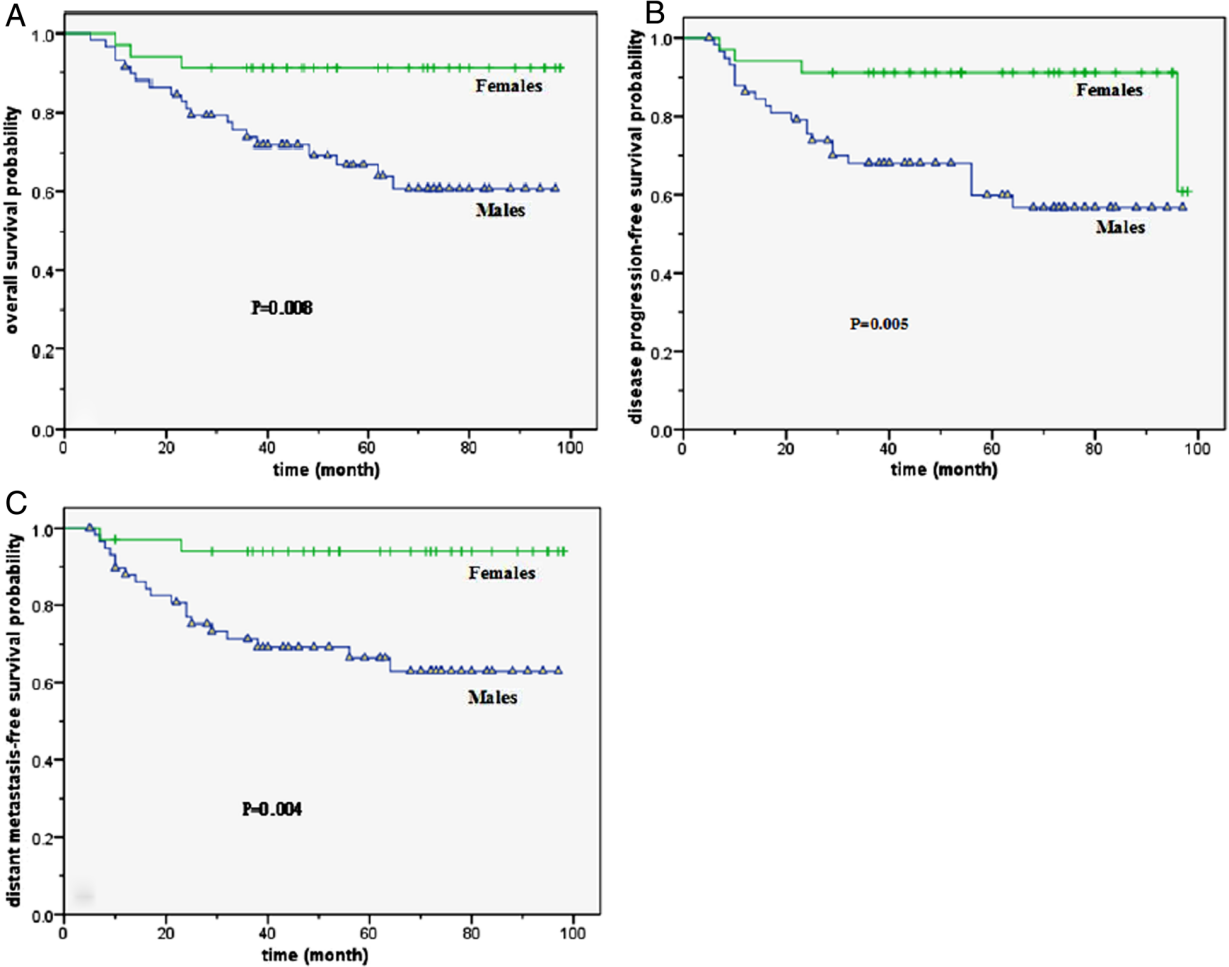
**Survival curves for male and female patients younger than 45 years old. A.** The overall survival curves of male and female. **B.** The disease progression-free survival curves of male and female. **C.** The distant metastasis-free survival curves of male and female.

**Figure 3:**
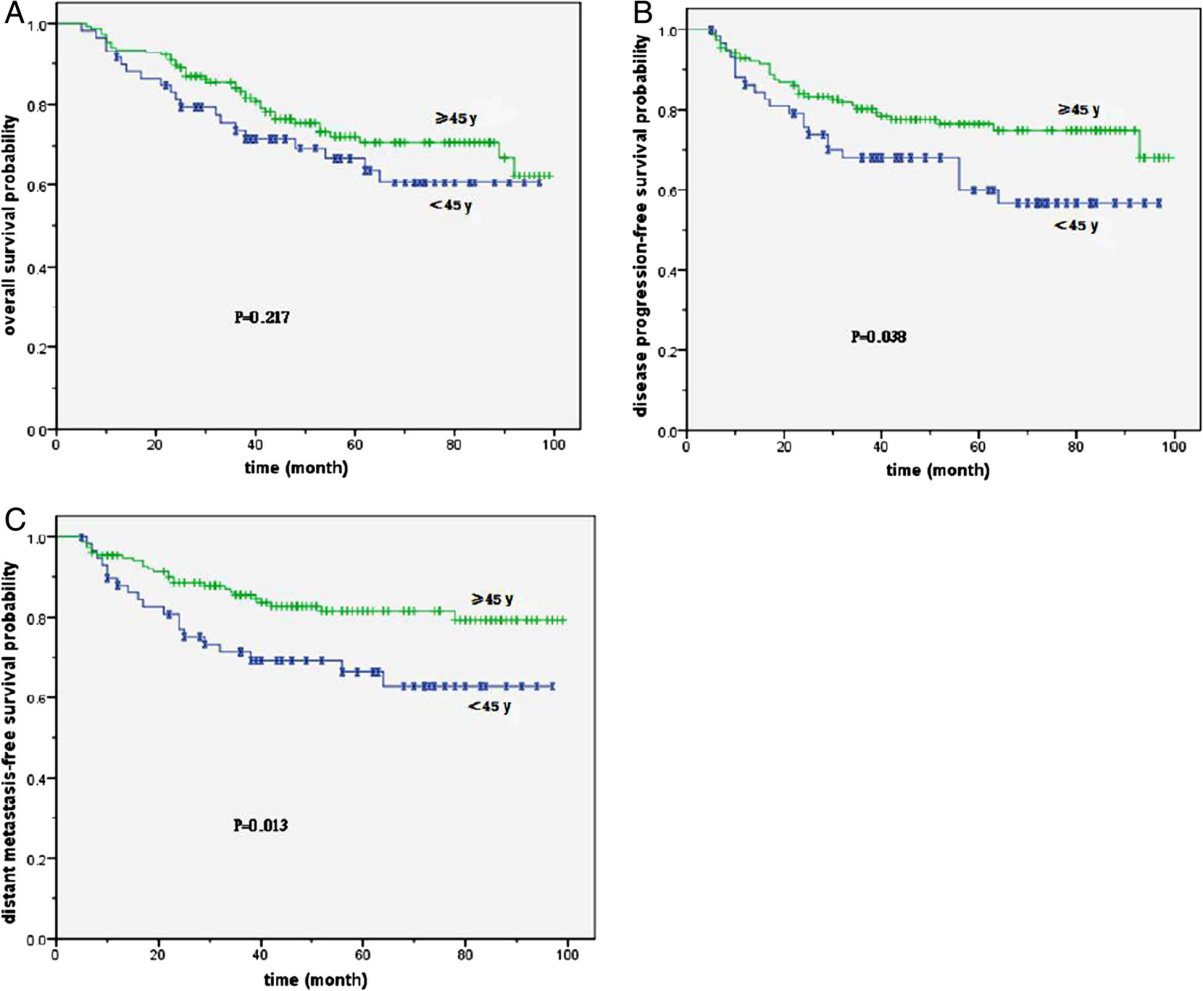
**Survival curves for male patients in different age groups. A.** The overall survival curves for male of different age groups. **B.** The disease progression-free survival curves for male of different age groups. **C.** The distant metastasis-free survival curves for male of different age groups.

For patients younger than 45, the male patients had a significantly unfavorable 5-year OS than female patients (66.8% compared to 91.2%, P = 0.008, *X*^2^ = 7.067), DPFS (59.9% compared to 91.2%, P = 0.005, *X*^2^ = 7.724) and DMFS (66.4% compared to 94.0%, P = 0.004, *X*^2^ = 8.461) (Figure 2). For patients older than 45, only the 5-year OS (72.2% compared to 96.0%, P = 0.001, *X*^2^ = 10.186) was significantly different. The 5-year DPFS (76.4% compared to 84.1%, P = 0.483, *X*^2^ = 0.493) and DMFS (81.5% compared to 86.3%, P = 0.677, *X*^2^ = 0.174) were not significantly different. Male patients younger than 45 had a lower 5-year DPFS (56.7% compared to 76.4%, P = 0.038, *X*^2^ = 4.324) and DMFS (62.9% compared to 84.5%, P = 0.013, *X*^2^ = 6.114) than older male patients. However, the gap of OS curves narrowed in both age groups for male patients. The survival difference was not statistically significant (Figure 3). In contrast, female patients showed no age-related differences for the 5-year DPFS (91.2% compared to 84.1%, P = 0.198, *X*^2^ = 1.656), DMFS (94.0% compared to 86.3%, P = 0.161, *X*^2^ = 1.969) and OS (91.2% compared to 96.0%, P = 0.346, *X*^2^ = 0.887).

The chemotherapy, including concurrent chemoradiation, neoadjuvant chemotherapy and adjuvant chemotherapy, did not improve the local-regional control rate or the distant metastasis rate. Although the patients with stage T3-4 who received concurrent chemoradiation had a tendency to improve the local failure rate (8/141 compared to 6/58), this was not a statistically significant difference.

The results of multivariate analysis on the prognostic factors are shown in Table 2. Gender and TNM stage, a measure of the extent of the disease, were independent prognostic factors on overall survival, disease progression-free survival and distant metastasis-free survival. Post-styloid parapharyngeal invasion and intracranial invasion were significant prognostic variables affecting local progression-free survival, whereas supraclavicular lymph node metastasis and visualization of extracapsular extension of the lymph node were identified as the main prognostic factors of regional progression-free survival.

**Table 2:** Multivariate analysis on the prognostic factors of survival

		**RR**	**95% CI**		***P***	***X***^***2***^
			**Lower**	**Upper**		
OS	Sex (male or female)	0.171	0.068	0.427	0.000	14.274
	TNM Stage	1.915	1.125	3.260	0.017	5.723
	Intracranial invasion	3.850	1.867	7.940	0.000	13.324
	Supraclavicular LNM	2.458	1.149	5.257	0.020	5.375
	LN extracapsular extension	3.183	1.854	5.464	0.000	17.642
DPFS	Sex (male or female)	0.499	0.277	0.900	0.021	5.331
	TNM Stage	2.320	1.530	3.517	0.000	15.702
	LN extracapsular extension	2.411	1.419	4.099	0.001	10.573
	LN necrosis	1.748	1.043	2.928	0.034	4.501
DMFS	Sex	0.483	0.243	0.961	0.038	4.302
	TNM stage	2.200	1.383	3.499	0.001	11.081
	LN extracapsular extension	3.680	2.115	6.405	0.000	21.244
LPFS	Post-styloid Parapharyngeal invasion	2.852	1.077	7.549	0.035	4.452
	Intracranial invasion	5.316	1.613	17.521	0.006	7.536
RPFS	Supraclavicular LNM	5.415	1.363	21.504	0.016	5.762
	LN extracapsular extension	5.148	1.450	18.270	0.011	6.427

*OS* = overall survival, *DPFS* = disease progression-free survival, *DMFS* = distant metastasis-free survival, *LPFS* = local progression-free survival, *RPFS* = regional progression-free survival, *LN* = lymph node, *LNM* = lymph node metastasis.

## Discussion

To date, only a few studies have focused specifically on the prognostic impact of gender and age on NPC. In addition, many reports studying the main impact of other prognostic factors have reported controversial results with regard to the prognostic impact of gender. Several larger Chinese studies [2-6] demonstrated that being female was a favorable factor for overall survival by multivariate analysis, although many studies found no significant difference in survival based on gender [7-9]. Some studies showed that gender affects other endpoints of prognostic analysis significantly, such as disease-free survival [2,5,10], local control [4,11,12], and distant metastasis [2,3,11]. Being male was an adverse prognostic factor in these studies.

We also found that male patients were more likely to have distant metastases than female patients and exhibited inferior overall survival and disease progression-free survival rates. Even in the subgroup analysis, similar results were obtained. The most intriguing result of our study was the male survival curves were consistently below female patients regardless of the statistical significance of the difference. If the sample size is big enough, maybe gender difference will become statistical significant for more end points. Furthermore, male patients exhibited a shorter interval before therapeutic failure compared to female patients. This suggests that a biological difference in tumor behavior exists between male and female patients. Gender differences in the incidence and prognosis of NPC may be due to genetic variants affected by the hormonal environment. Nasr [13] was the first to report on functional VEGF polymorphisms in patients with NPC in 2008. He found that men carrying the VEGF-2578 C allele had higher risk for NPC than women. Furthermore, there was a significant association between the distribution of the VEGF-2578 CC genotype among male patients and a larger tumor size and advanced cancer stage.

Most of the results studying the prognostic impact of age in NPC indicate that younger patients have a higher overall survival [2,3,5,10,12]. Some studies also showed that disease-related survival [2,5,10,11], local control [2,10] and distant metastasis [2] were affected by age. Only Ma [3] has reported that patients younger than 40 have lower OS and local control rates by multivariate analysis. However, these studies were not adjusted to account for other prognostic factors, such as tumor size, stage, therapeutic strategies and performance status. Because older patients were more likely to have comorbidities and a poorer performance status, which may contribute to a low tolerance for intense treatment (radiotherapy and/or chemotherapy), there were more non-tumor-related deaths in older patients. Moreover, the vast majority of previous studies employed conventional radiotherapy technology. The radiotherapy technique impacts the dose delivered to the local lesion and could pose a tolerance problem, affecting tumor control and survival in patients. Intensity-modulated radiotherapy offers the potential for improved treatment outcomes because patients, including older patients, have a high tolerance for the therapy. Furthermore, there were more opportunities to find differences in biological behavior between male and female patients due to the longer follow-up period. Our data showed that 5-year OS, DPFS and DMFS were more than 20% lower for male patients younger than 45 compared with female patients of the same age. Indeed, younger male patients had 20% lower DPFS and DMFS values than older male patients. These data suggest that the variables of both gender and age must be considered important predictors for survival.

There are numerous reports concerning gender differences on the prognosis of other malignant tumors. An Asian population-based study observed that female patients display better survival rates for the majority of solid tumor sites, even after adjustment for age and stage. However, the survival gaps between male and female narrow as age increases [14]. Joosse [15] reported that the prognostic outlook for female patients compared to male patients with melanoma was equal in the 60-year-old age group, unlike the female patient advantage observed in the 45-year-old group, when examining the end points of relapse-free survival, time to lymph node metastasis, and time to distant metastasis. Wolf et al. [16] found that the favorable prognostic effect seen in female patients with small cell lung cancer was restricted to patients younger than 60 years old.

It is well known that sexual hormone levels begin to decrease on average at the age of forty to fifty years old and reach their lowest values at sixty years old. Some hypotheses concerning the cause of gender differences have not been confirmed, in part due to the particular focus placed on the effect of estrogen [15,17,18]. In fact, several preclinical studies had demonstrated that androgens can induce proliferative changes in cancer cell lines and promote tumorigenesis in animal models by androgens receptor (AR) [19,20] Maasberg et al. [21] (1989) found that application of testosterone resulted in a 3-fold increase in cell proliferation in a cloning assay, whereas estrogen had no influence on tumor growth. This growth stimulation could be counteracted by the addition of anti-androgens such as cyproterone acetate or flutamide. These *in vitro* experiments imply that sexual hormones may play a role in the regulation of tumor growth. Our study demonstrated that being a younger male patient corresponded to a less favorable prognosis and suggested that male sexual hormones like androgens might drive tumor development in NPC.

The limitation of this paper is that it is a retrospective analysis. The underlying causes of the difference in the behavior of NPC between genders are not completely known. However, it is certain that male patients had a significantly unfavorable 5-year OS, DPFS and DMFS. In particular, younger male patients had more than 20% lower survival rates than female patients. Gender is a strong independent prognostic factor for NPC. Future work should focus on researching the mechanisms of gender difference in NPC progression and confirm the relationship between gender and genetic polymorphisms in experimental and clinical studies.

## Conclusion

Gender and age are strong independent prognostic factors for NPC in this study. Younger men were more likely to have distant metastases and exhibited inferior overall survival and disease progression-free survival rates.

## Competing interests

The authors declare that they have no competing interest.

## Authors’ contributions

*All authors substantially contributed to the current manuscript as listed below*. GX drafted the manuscript, is the principal investigator of the study and designed this study. The rest of the authors collected and reviewed data. All authors read and approved the final manuscript.

## Pre-publication history

The pre-publication history for this paper can be accessed here:

http://www.biomedcentral.com/1471-2407/13/226/prepub
